# Human Milk Glucocorticoid Levels Are Associated With Infant Adiposity and Head Circumference Over the First Year of Life

**DOI:** 10.3389/fnut.2020.00166

**Published:** 2020-09-11

**Authors:** Shikha Pundir, Zoya Gridneva, Avinesh Pillai, Eric B. Thorstensen, Clare R. Wall, Donna T. Geddes, David Cameron-Smith

**Affiliations:** ^1^Liggins Institute, The University of Auckland, Auckland, New Zealand; ^2^School of Molecular Sciences, The University of Western Australia, Perth, WA, Australia; ^3^Department of Statistics, The University of Auckland, Auckland, New Zealand; ^4^Faculty of Medical and Health Science, The University of Auckland, Auckland, New Zealand; ^5^Singapore Institute for Clinical Sciences, Agency for Science, Technology and Research (A^*^STAR), Singapore, Singapore

**Keywords:** cortisol, cortisone, lactation, mass spectrometry, fat mass, head circumference

## Abstract

Human milk (HM) is a complex and dynamic biological fluid, which contains appreciable concentrations of the glucocorticoids, cortisol and cortisone. Experimental studies in non-human primates suggest the HM glucocorticoids' impact on infant growth and body composition. In this current study, analysis is made of the relationships between HM glucocorticoid concentrations and the infant growth and development over the first year of life. HM was collected by lactating healthy women (*n* = 18), using a standardized protocol, at 2, 5, 9, and 12 months after childbirth. Cortisol and cortisone concentrations in the HM were measured using liquid chromatography mass spectrometry. Infant weight, length and head circumference were measured by standard protocols and percentage fat mass (% FM) determined by whole body bioimpedance. Cortisol and cortisone concentrations were unaltered over the analyzed lactation period (2–12 months), and were altered by infant sex. Although, HM cortisol was positively associated with infant percentage fat mass (% FM) (*p* = 0.008) and cortisone positively associated with infant head circumference (*p* = 0.01). For the first 12 months of life, the concentration of HM glucocorticoids levels was positively associated with infant adiposity (%FM) and head circumference. This preliminary evidence provides insight to a possible relationship between ingested HM glucocorticoids and infant body composition. Further studies are required to determine the mechanisms regulating HM glucocorticoids.

## Introduction

Nutrition during the first 1000 days of life, a period from conception to the child's second year, has a major impact on the infant's growth and development (1). It is during this critical period of life where subtle changes in growth and developmental trajectories can have substantial impact on the health of that individual later in life, including obesity and non-communicable disease risks through childhood and into adulthood (2, 3). Numerous studies demonstrate that the infant intake of mothers' milk (human milk: HM) and establishment of breastfeeding for longer periods confer benefits for both the mother and infant, from nourishment, cognitive benefits, immune protection and reduces the risk of childhood obesity (4–8).

Human milk (HM) is complex and dynamic, not only containing nutritive factors (9), but also being recognized as a rich source of hormones, which have been demonstrated to impact directly or indirectly on infant body functions (10). The presence of hormones, such as leptin, insulin, ghrelin, adiponectin, and insulin like growth factor-1 (IGF-1) is of great importance, due to their involvement in the key aspects of appetite and metabolic regulation (10–13). Thus, amoungst infants where HM is the predominant source of nourishment for the first year of life, subtle variation in HM composition, may modify early growth, and development.

One major class of hormones present in HM are the glucocorticoids, cortisol, and cortisone. All are involved in the regulation of metabolic homeostasis and inflammation (14). However, far less is known of the potential biological functions of HM derived glucocorticoids in the developing infant. It has been demonstrated that the glucocorticoids are predominantly transferred from the maternal circulation into HM. Unlike plasma, the concentration of cortisone in HM is higher than cortisol and also greater than the levels measured in the maternal plasma circulation (15–17). Furthermore, differences exist in salivary cortisol concentrations between breastfed infants and formula feed infants (18) and plasma cortisol strongly correlates with HM cortisol over the first year of life (15). Whilst the role of HM cortisol has not been fully elucidated, HM cortisol correlates with infant mood and aspects of behavioral development, including sex specific temperament issues (19, 20). Hahn et al. (21) demonstrated that higher HM cortisol was associated with lower body mass index percentile (BMIP) at 2 years of age, suggesting that HM cortisol is protective against later life excess adiposity. However, it is difficult to examine closely the impacts of HM glucocorticoid composition and growth and body composition in toddlers, given the increased role exerted by increasing solid food and supplemental milk, including varying formula based beverages on the childs development (22). Therefore, studies are still required in younger children.

Much is known about the nutritional composition of HM and its dynamicity, to meet the changing demands of infants at every stage of lactation (9, 23, 24). Studies have provided considerable details of the changing HM macronutrients over the first 12 months of lactation (25). There remains little data on the variation and regulation of HM glucocorticoids throughout lactation. To the best of our knowledge, there has been only one human study (26) that has analyzed the concentrations of cortisol over an extended period of 12 months of lactation. In this study, HM samples were analyzed to determine the changes in HM cortisol measured by radioimmunoassay and corticosteroid binding globulin (CBG) assay at different stages of lactation, largely between late pregnancy and after the cessation of breastfeeding, reporting lower concentrations during the established lactation (26). However, this study did not quantify cortisone, the predominant glucocorticoid in HM, nor did it examine the relationship between HM glucocorticoids and infant growth. Therefore, in the current study, we aimed to measure the concentration of HM cortisol and cortisone using liquid chromatography mass spectrometry (LC-MS/MS), and investigate their relationships with the development of breastfed infant body composition and growth over the first 12 months of life.

## Methods

### Study Design and Subjects

Between 2013 and 2015, breastfed infants (*n* = 18) of predominantly Caucasian and mothers of higher social-economic status were recruited from the community, primarily via the Australian Breastfeeding Association, Perth, Western Australia. Inclusion criteria were: healthy singletons, gestational age ≥ 37 weeks, exclusively breastfed at 2 and 5 months (27), and maternal intention to breastfeed until 12 months, without the introduction of formula. Exclusion criteria were: infant factors that could potentially influence growth and development of BC, maternal smoking and low milk supply. All mothers provided written informed consent to participate in the study, which was approved by The University of Western Australia Human Research Ethics Committee (RA/1/4253, RA/4/1/2639) and registered with the Australian New Zealand Clinical Trials Registry (ACTRN12616000368437).

Maternal and infant anthropometric measurements were made at the time of sample collection. Participants visited the research laboratory at King Edward Memorial Hospital for Women (Subiaco, Perth, WA) for up to 4 monitored breastfeeding sessions between March 2013 and September 2015. At each study session, the infants were weighed pre-feed, and then the mother breastfed her infant. Infant bioelectrical spectroscopy (BIS) measurements were taken pre-feed, unless impractical, then they were taken post-feed (28). Anthropometric measurements were taken post-feed. Clothing was removed for the measurements except for a dry diaper and a singlet.

### Anthropometric Measurements

Infant's weight was determined before breastfeeding using Medela Electronic Baby Weigh Scales (±2.0 g; Medela Inc., McHenry, IL, USA), whereas maternal weight was measured using Seca electronic scales (±0.1 kg; Seca, Chino, CA, USA). Maternal and infant BMI was calculated as Bodyweight (kg)/(Height (m))^2^. Infant crown-heel length was measured once to the nearest 0.1 cm using non-stretch tape and a headpiece and a foot piece, both applied perpendicularly to the hard surface. Infant head circumference was measured with a non-stretch tape to the nearest 0.1 cm. Maternal height measured against a calibrated marked wall (accuracy~0.1 cm).

#### Bioimpedance Spectroscopy Measurements

Whole body bioimpedance (wrist to ankle) of infants and mothers was measured using a bioelectrical impedance analyzer (ImpediMed SFB7, Brisbane, Queensland, Australia). Mothers were measured in a supine position on a non-conductive surface. A series of ten consecutive measurements of percentage fat mass (% FM) were taken within 1–2 min and averaged for data analysis. Within the participant coefficient of variation (CV) for maternal % FM was 0.21% (29). Infants' whole body bioimpedance was measured by applying an adult protocol as used previously with data analyzed using settings customized for infants (30, 31). Values of resistance (ohm) at a frequency of 50 kHz (R_50_) were determined from the curve of best fit, averaged for analysis purposes and used in BIS age-matched equations for fat-free mass. BIS-based prediction equations for infant BC (31–33) were sourced from the literature, evaluated compared with the reference data (30) and selected according to the following criteria: the absence of significant difference from the reference distribution, closest age match, predominantly Caucasian population. Within participant CV for infant R_50_ was 1.5% (28).

### Milk Collection

All sample sets with the exception of one were collected between 9:30 and 10:30 am at the time of measurements at King Edward Memorial Hospital for Women (Perth, Western Australia) at 2 and/or 5, 9, and 12 months postpartum. Small (1–2 mL) pre- and post-feed milk samples were collected into polypropylene 5 mL polypropylene vials (Disposable Products, Adelaide, Australia) and frozen at −20°C. Samples were shipped on dry ice to The University of Auckland, Auckland, New Zealand for glucocorticoid analysis, and were subsequently kept frozen at −80°C until analysis.

Milk samples were divided into four intervals and were classified as T2 (samples collected between 1.9 and 2.3 months) (13 milk samples), T5 (samples collected between 4.8 and 5.5 months) (18 milk samples), T9 (samples collected between 8.8 and 9.8 months) (18 milk samples) and T12 (samples collected between 11.6 and 12.7 months) (13 milk samples). These time periods were used in the subsequent analysis as four major time points. Milk samples were analyzed for all participants on a minimum of 3 timepoints.

## Human Milk Glucacorticoid Analysis

### Sample Preparation

HM steroids were measured by liquid chromatography mass spectrometry (LC-MS), as described previously (17). The internal standard consisted of 12 ng/ml cortisol d4, 60 mg/ml corticosterone d8, prepared in water. All milk samples were warmed to 37°C and vortexed for 10 s before 100 μl of the sample was added to a glass tube containing 100 μL of the internal standard solution. Steroids were then extracted using 1 ml ethyl acetate (Merck, Germany); the top organic layer was removed into a separate tube and then vacuum dried (Savant, SC250EX, Thermo Scientific, USA) for ~1 h. The dried residues were reconstituted with 80 μl of 50% methanol (Merck, Germany)/water and transferred to HPLC injector vials. All samples were run in duplicate, and average values are reported.

### Liquid Chromatography Tandem Mass Spectroscopy

The HPLC tandem mass spectrometer (MS) used an Accela MS pump and auto sampler followed by an Ion Max APCI source on a Thermo Scientific Quantum Ultra AM triple quadrupole mass spectrometer, all controlled by Finnigan Xcaliber software (Thermo Electron Corporation, San Jose, CA.). The mobile phase was a methanol-water gradient starting at 60:40(v/v) (peaking at 80:20 before returning back to 60:40) at 300 μl/min. The chromatography was performed using a Phenomenex Luna C18 (2)-HST column (100 × 3 mm, 2.5 μm particle size) at 40°C. The instrument was set up in selective reaction monitoring (SRM) mode with the following mass transitions: m/z 363.2 → 121.09 for cortisol, 361.1 → 163.04 for cortisone, 367.1 → 121.04 for cortisol d4 and 355.2 → 125.10 for corticosterone d8. Dissociation voltage was 24V, and the collision gas (Argon) was set at 1.2 m Torr for all steroids. Steroid concentrations were calculated from a standard curve generated for each steroid relative to its internal standard (cortisol d4 for cortisol and corticosterone d8 for cortisone).

### Statistical Analysis

Results are expressed as mean ± SD unless mentioned otherwise. Normality was checked and normal distribution was observed. ANOVA was used to compare the differences in cortisol and cortisone concentration between different stages of lactation. Spearman correlation was run to assess the relationship between HM glucocorticoids and maternal and infant characteristics. Linear mixed models were employed to investigate associations between HM glucocorticoids concentration and both maternal and infant characteristics. Linear regression was performed at four timepoints to investigate associations with HM glucocorticoids. A *p* ≤ 0.05 was considered significant. There was no imputation carried out for missing values. Means and standard deviations were used for reporting. Statistical analysis was carried out using SPSS software (SPSS version 23.0 for windows, IBM SPSS Inc, IL USA) and GraphPad Prism 7.0 software was used for Figures (California, USA). Lattice plots were produced using R software version 2.15.2.

## Results

### Participants

The demographics and characteristics of the study participants are described in Table 1. Of 18 mothers, 16 were Caucasian and 2 Asian; 17 mothers were married; 15 mothers completed last year (year 12) of school, 12 indicated various diplomas as further education. Three mothers reported depression and one exhibited hypertension.

**Table 1:** Maternal (M) and infant (Inf.) characteristics of longitudinal study measuring the concentration of glucocorticoids in HM samples at T2, T5, T9, and T12 months postpartum.

	**Mean ± SD**			
Mothers (*n =* 18)				
Age (years)	33.88 ± 4.88			
Lactation stage (months)	T2.0	T 5.0	T 9.0	T 12.0
M weight (kg)	73.06 ± 17.35	62.79 ± 16.34	68.02 ± 18.66	65.83 ± 20.71
M %FM (BIS)	35.04 ± 5.27	32.21 ± 7.07	31.68 ± 7.24	28.97 ± 7.47
Infant sex	Female (*n =* 8)		Male (*n =* 10)	
Inf. Length (cm)	57.64 ± 1.95	64.76 ± 2.35	70.25 ± 2.05	74.15 ± 2.43
Inf. Weight (kg)	5.59 ± 0.88	7.46 ± 0.98	8.76 ± 0.95	9.66 ± 0.79
Inf. BMI	16.30 ± 1.38	17.74 ± 1.70	17.59 ± 1.58	17.30 ± 1.116
Inf. Head circum. (cm)	39.62 ± 1.35	42.91 ± 1.72	45.45 ± 1.68	46.32 ± 1.42
Inf. %FM (BIS)	21.74 ± 2.17	28.24 ± 3.26	25.13 ± 4.65	24.26 ± 3.35

*BIS, bioelectrical impedance spectroscopy; BMI, body mass index; %FM, percentage fat mass; T2 (1.9–2.3 months), T5 (4.8–5.5 months), T9 (8.8–9.8 months), and T12 (11.6–12.7 months)*.

All 18 infants were exclusively breastfed at 2 and 5 months and continued to breastfeed at 9 months. Fifteen infants (83%) continued to breastfeed on demand at 12 months. One male infant ceased breastfeeding 8 days before the 12-month appointment, one male infant was being weaned at the time of 12-month appointment, no samples were provided; one female infant stopped at 10 months after birth; these three infants were measured at the time of the 12-month appointment. One male infant was sick and did not attend the last appointment.

Infant % FM (*p* < 0.00) changed significantly with an increase from 2 to 5 months, followed by a decrease at 9 and 12 months.

### Changes in HM Glucocorticoid Concentration Throughout the Year

**Figure 1:**
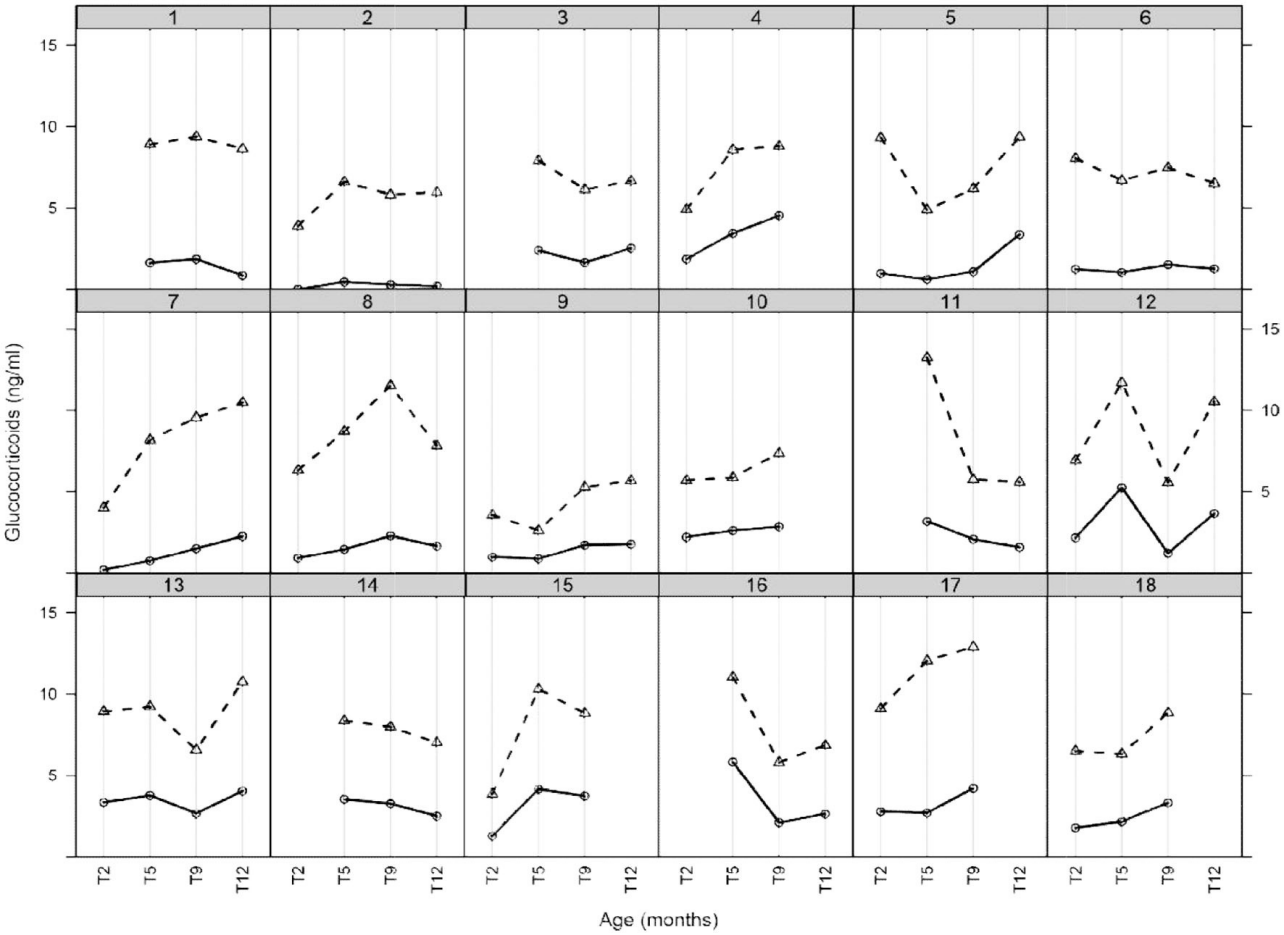
HM glucocorticoids (cortisol and cortisone) during the first 12 months of established lactation. Each box of the lattice plot indicates a single mother milk glucocorticoid profile with symbol (o) representing cortisol and (Δ) representing cortisone. Each solid line indicates cortisol and dashed line cortisone. Time points are categorized as T2 (1.9–2.3 months), T5 (4.8–5.5 months), T9 (8.8–9.8 months), and T12 (11.6–12.7 months).

The concentration of cortisol and cortisone in each individual and at each time point is shown in Figure 1. The concentration of cortisol ranged between 0.01 and 5.82 ng/ml and cortisone ranged between 2.60 and 13.23 ng/ml (Table 2). For all analyzed samples, cortisone was the predominant glucocorticoid at all the four time points. Mean concentrations of cortisol (*p* = 0.10), cortisone (*p* = 0.06) and cortisol/cortisone ratio (*p* = 0.39) did not differ significantly over the period of 12 months.

**Table 2:** Summary of glucocorticoids in HM samples (n = 63) collected from 18 breastfeeding Western Australian mothers at different stages of lactation (2, 5, 9, and 12 months).

	**Mean ± SD (ng/ml)**			
Lactation stage (months)	T2.0	T5.0	T9.0	T12.0
Cortisol (ng/ml)	1.52 ± 0.96	2.68 ± 1.65	2.32 ± 1.13	2.18 ± 1.11
Cortisone (ng/ml)	6.19 ± 2.00	8.57 ± 2.72	7.76 ± 2.15	7.83 ± 1.90
Cortisol/cortisone ratio	0.22 ± 0.13	0.30 ± 0.13	0.29 ± 0.11	0.27 ± 0.11

### Relationships Between Maternal Characteristics and HM Glucocorticoids

HM cortisol (*r*_*s*_ = −0.29, *p* = 0.02), cortisone (*r*_*s*_ = −0.27, *p* = 0.03) and cortisol/cortisone ratio (*r*_*s*_ = −0.24, *p* = 0.05) showed associations with maternal height. Furthermore, an overall significant positive correlation was found between HM cortisol/cortisone ratio and maternal BMI (*r*_*s*_ = 0.33, *p* = 0.009). Maternal % FM showed no correlation with cortisol (*r*_*s*_ = 0.21, *p* = 0.09) and cortisol/cortisone ratio (*r*_*s*_ = 0.24, *p* = 0.06). The regression analysis identified a positive relationship between HM cortisol and maternal height (*p* = 0.02), and maternal BMI (*p* = 0.04). However, no association was found between HM cortisone and maternal BMI (*p* = 0.81) or maternal height (*p* = 0.60).

### Relationships Between Infant Characteristics and HM Glucocorticoids

Overall, a weak positive correlation was found between cortisol and infant head circumference (*r*_*s*_ = 0.25, *p* = 0.05) and % FM (*r*_*s*_ = 0.27 *p* = 0.03). Furthermore, the HM cortisol/cortisone ratio showed a positive correlation with infant % FM (*r*_*s*_ = 0.34 *p* = 0.01) and BMI (*r*_*s*_ = 0.28, *p* = 0.032), while cortisone showed no significant associations with any of the infant parameters (*p* = 0.07, head circumference, *r*_*s*_ = 0.23). The mixed model analysis showed a positive relationship between HM cortisol and infant % FM (*p* = 0.008), and head circumference (*p* = 0.05); and showed no associations with infant length (*p* = 0.37), weight (*p* = 0.56), and BMI (*p* = 0.26). Whereas, cortisone showed a significant positive association with infant head circumference (*p* = 0.01) and showed no association with infant % FM (*p* = 1.00), length (*p* = 0.61), weight (*p* = 1.00), and BMI (*p* = 1.0). Furthermore, cortisol/cortisone ratio showed a significant association with infant % FM (*p* = 0.04) and no associations were found between cortisol/cortisone ratio and infant length (*p* = 0.50), weight (*p* = 0.81), BMI (*p* = 0.72), and head circumference (*p* = 0.11). Follow-up analysis using linear regression (Table 3) established that infant % FM and head circumference significantly associated with cortisol (PE+/–SE; *p* = 0.05) and cortisone (PE+/–SE; *p* = 0.03) in HM, respectively.

**Table 3:** Linear regression: associations between milk glucocorticoids and infant characteristics.

**Infant factors**	**Unstandardized β**	**Standardized β**	**SE**	***p***
Infant percentage fat mass (with cortisol)	0.08	0.24	0.04	0.05
Infant head circumference (with cortisone)	0.21	0.26	0.10	0.03

## Discussion

The concentrations of the glucocorticoid hormones in HM, cortisol and cortisone, exhibited unique individual variation at the measured timepoints during the first year on life. Averaged across the sampled cohort, there was no consistent pattern in the HM glucocorticoid concentrations throughout the first year of lactation. However, this study providing evidence that higher concentrations of HM cortisol were positively and significantly related to greater infant adiposity (% FM). Higher concentrations of HM cortisone were positively associated with larger infant head circumferences. These data provide preliminary evidence that HM glucocorticoids exert influence on infant growth and body composition. HM cortisol was correlated with maternal BMI and height, but not HM cortisone, suggesting a more complex relationship between HM glucocorticoids and maternal adiposity.

The composition of HM is influenced by the complex interplay between maternal, infant and diverse environmental factors (10); and also varies depending upon the stages of lactation (34–36). In the current study, it was demonstrated that cortisol, cortisone and their ratio did not significantly change over the first 12 months of lactation. These data further confirm the prior demonstration that HM cortisol remained unaltered between 1 and 12 months of lactations (26). Interestingly, the apparent concentration of cortisol measured in this earlier study (26) ranged between 0.2 and 32 ng/ml, which was on average, 5.5-fold higher than that of the current study. Given the quantitative nature of the current LC-MS technique and the ability to accurately discriminate between differing glucocorticoids, it is then likely that the previously used immunoassay may have exaggerated the abundance, potentially due to antibody cross-reactivity.

In this study, HM cortisol is significantly associated with adiposity (% FM) in infants, with higher levels of HM cortisol being associated with greater infant adiposity (over the first 12 months of lactation). Elevated circulatory cortisol is known to be a potent stimulator of body fat mass gain and the mechanisms are well-described (37, 38). However, majority of these studies have been done in adults with metabolic syndrome and hence require more robust studies on the long term effect of glucocorticoids on the breastfeed infants. A recent study by Hahn et al. (21) provided prior evidence that HM cortisol is the predictor of infant obesity. In this study, infants exposed to higher milk cortisol concentration exhibited reduced infant body mass index percentile (BMIP) at 2 years of age. Furthermore, they reported that the milk cortisol did not predict weight gain, but infants exposed to elevated level of cortisol grew taller, causing the variations in BMIP. However, not much is known about the impact of milk-ingested glucocorticoids, particularly cortisoneThis study did not examine the relationship of HM cortisone. Unlike cortisol, cortisone is the biologically inactive steroid, requiring type 1-11β-hydroxysteroid dehydrogenase (11 βHSD) enzyme conversion into physiologically active cortisol and 11 βHSD type-2 for converting cortisol into cortisone (39). Since cortisone is not secreted in measurable amounts in maternal plasma, 11 βHSD type-2 could potentially be present within the milk or mammary gland. However, the function and mechanisms of this conversion in milk remains largely unknown.

During infancy or early childhood, head circumference is commonly used as an indicator of infant brain size (40) and is also used as a proxy for infant's neurological development, cognitive function and intracranial volume (41). Numerous prospective studies have shown that chronic prenatal maternal stress during pregnancy influences infant brain development and is associated with smaller head circumference, although the underlying mechanisms are unclear (42–46). Head circumference is an old method of measuring infant brain development, but still is considered reliable. Dupont el al. (47) investigated the predictive value of head circumference during the first year of life on early child development. They demonstrated that post-natal head circumference growth positively predicted gross motor skills as well as behavioral growth at 2 years of age. However, only a few studies have investigated the relationship between maternal perceived stress and infant neurodevelopmental outcomes (48). Studies examining these associations have pronounced mixed results in humans; some found a negative association between maternal stress and infant head circumference (45, 49), while others failed to show any association (43). Chronic prenatal stress disrupts cognitive performance and reduces brain volume in the area related to learning and memory (44). A negative association between prenatal maternal stress and fetal head growth development suggests a key role of maternal stress in regulating fetal head growth (45). Recently the role of glucocorticoids has become evident in infant development (50, 51), and in this current study, we identified that increasing head circumference could be associated with HM glucocorticoids. Although, correlation does not imply causation, hence results should be read with caution.

An association between early glucocorticoid exposure and infant metabolism is complex and regulated by many maternal related factors such as maternal circulating levels. Excessive exposure to both endogenous and exogenous glucocorticoids during pregnancy and lactation have been previously linked to obesity risk factors (52, 53). This study has shown a significant association between maternal BMI and HM cortisol levels. There are several factors that contribute to the plasticity of HM, including maternal BMI, an important contributor to the hormonal profile of HM, further affecting the developmental trajectories of HM fed infants (29). Evidence suggests that individuals with higher BMI are more likely to have an increased level of circulating cortisol (54, 55). This study is the first to demonstrate an association between maternal BMI and HM cortisol concentration. This is in accordance with previous studies that report obesity or higher BMI to be an indicator of increased circulatory or saliva cortisol level (56, 57).

A limitation of the current study was the absence of maternal plasma samples. Therefore, this study is unable to report on possible relationships between maternal hypothalamic variabilities and the HM glucocorticoids. Furthermore, the sample set was small and predominately from Caucasian women. Further studies examining HM hormonal profiles and infant outcomes, at different stages of lactation, will further elucidate the possibilities of lactocrine programming.

In conclusion, HM cortisol and cortisone demonstrated unique variability in each women, but on average remained constant throughout the first 12 months of lactation. HM cortisol and cortisone were positively correlated with infant head circumference and % FM during the first year of life. This current research points to the need to better understand the determinants of HM glucocorticoids regulation. Yet the implications the HM glucocorticoids function to regulate infant metabolism, appetite and behavior suggest that further exploration is required to better understand how ingested hormones can mediate these peripheral actions.

## Data Availability Statement

The raw data supporting the conclusions of this article will be made available by the authors, without undue reservation.

## Ethics Statement

The studies involving human participants were reviewed and approved by The University of Western Australia Human Research Ethics Committee. The patients/participants provided their written informed consent to participate in this study.

## Author Contributions

SP conducted the analyses, drafted the initial manuscript, reviewed, and revised the manuscript. All authors were responsible for the idea conception, approved the final manuscript as submitted and agree to be accountable for all aspects of the work.

## Conflict of Interest

SP and ET are employed through Liggins Institute, The University of Auckland. DC-S was an employee of the Liggins Institute when the study was conducted. CW is employed through the Faculty of Medical and Health Science, The University of Auckland. AP is employed through the University of Auckland. DG and ZG receive a salary from an unrestricted research grant from Medela AG (Switzerland) administered by The University of Western Australia. Medela AG had no role in the design of the study; in the collection, analyses, or interpretation of the data; in the writing of the manuscript, and in the decision to publish the results.
